# Difficulties encountered by public health workers in COVID-19 outbreak: a cross-sectional study based on five provinces

**DOI:** 10.1186/s12913-021-06699-4

**Published:** 2021-07-05

**Authors:** Zhicheng Du, Hua You, Huan Zhou, Xiaohui Wang, Jingdong Xu, Yan Li, Shan Li, Lina Ma, Jing Gu, Yuantao Hao

**Affiliations:** 1 School of Public Health, Global Health Institute, Key Laboratory of Tropical Disease Control, Sun Yat-sen University, 510080 Guangzhou, China; 2 School of Public Health, Nanjing Medical University, 210000 Nanjing, China; 3 West China School of Public Health, West China Fourth Hospital, Sichuan University, 610000 Chengdu, China; 4 School of Public Health, Lanzhou University, 730000 Lanzhou, China; 5 Hubei Provincial Center for Disease Control and Prevention, 430097 Wuhan, China; 6 Guangzhou Center for Disease Control and Prevention, 510440 Guangzhou, China; 7 Zigong Center for Disease Control and Prevention, 643000 Zigong, China

**Keywords:** Public health workers, Difficulties, COVID-19

## Abstract

**Objectives:**

The aim of this study is to address the difficulties encountered by public health workers in the early and middle stages of their efforts to combat COVID-19, compare the gaps among different types of institutions, and identify shortcomings in epidemic control.

**Methods:**

Using multi-stage sampling, a survey of public health workers involved in the prevention and control of COVID-19 was conducted from 18 February to 1 March 2020 through a self-administered questionnaire. These public health workers were from the primary health care center (defined as “primary-urban” and “primary-rural” for those in urban and rural areas, respectively) and the center for disease control and prevention (defined as “non-primary”) in five provinces including Hubei, Guangdong, Sichuan, Jiangsu and Gansu, China.

**Results:**

A total of 9,475 public health workers were surveyed, of which 40.0 %, 27.0 % and 33.0 % were from the primary-rural, primary-urban and non-primary, respectively. The resources shortage were reported by 27.9 % participants, with the primary-rural being the worst affected (*OR* = 1.201, 95 %*CI*: 1.073–1.345). The difficulties in data processing were reported by 31.5 % participants, with no significant differences among institutions. The difficulties in communication and coordination were reported by 29.8 % participants, with the non-primary being the most serious (primary-rural: *OR* = 0.520, 95 %*CI*: 0.446–0.606; primary-urban: *OR* = 0.533, 95 %*CI*: 0.454–0.625). The difficulties with target audiences were reported by 20.2 % participants, with the primary-urban being the worst (*OR* = 1.368, 95 %*CI*: 1.199–1.560). The psychological distress were reported by 48.8 % participants, with no significant differences among institutions.

**Conclusions:**

Psychological distress is the most serious problem in the prevention and control of COVID-19. Resources shortage in primary-rural, difficulties in communication and coordination in non-primary, and difficulties with target audiences in the primary-urban deserve attention. This study will provide scientific evidences for improving the national public health emergency management system, especially for reducing the urban-rural differences in emergency response capacity.

**Supplementary Information:**

The online version contains supplementary material available at 10.1186/s12913-021-06699-4.

## Introduction

As of 12th April 2020, a total of 82,160 cases of COVID-19 had been diagnosed in China, and 3,341 cases had died [1]. By the date of 1st April 2020, the spread of COVID-19 in China has been basically interrupted [2]. All aspects of society and economy have been put to an unprecedented and severe test. As of 13th March 2020, China’s investment in fighting the epidemic had reached 116.9 billion Chinese Yuan (≈ 16.9 billion US Dollar with the annual exchange rate in 2019 [3]), according to the State Council’s joint prevention and control mechanism press conference [4].

As the COVID-19 was caused by a new coronavirus and spread extremely fast, the outbreak spread across the world in just a few months, with the number of infections and deaths rising rapidly. The medical manpower and material resources at the time were almost unable to cope with the sudden pandemic. Healthcare systems in all countries were under strain. As of 7th April 2020, the total number of medical staff assisting Hubei, China from other provinces has reached more than 42,600 [5]. There are also countless public health workers, community workers and volunteers who work on the front line of outbreak prevention and control.

In China, the main institutions providing public health services are Centers for Disease Prevention and Control (CDC), Health Education Centers (HEC) and Community Health Centers/stations (CHC). In the rural areas, the township health centers and village health clinics assume the responsibilities of community health service centers/stations in the urban areas. China’s public health service system is a top-down system with multiple tiers. Of these, the city level and above are usually referred to as “non-primary”, and the level below city as “primary”. During the COVID-19 epidemic, the staff from the CDCs, HECs, and CHCs were mainly responsible for the prevention and control of the outbreak. These staff were defined as “public health workers” in our study, all of whom had a public health professional background or worked in public health and were paid by the government finance.

The public health workers are the important force in the fight against COVID-19 epidemic. They are responsible for carrying out zero-distance sampling and testing, epidemiological investigation, close contact tracing, outbreak analysis, disinfection of the public environment, and community prevention and control [6]. The COVID-19 epidemic, while a great stage victory, has also exposed shortcomings and deficiencies in our public health sectors. For example, President Xi pointed out that the first line of defence should be weaved tightly and firmly by strengthening primary capacity-building for prevention and control in rural and community areas. Some scholars have proposed that public health expenditure should be tilted towards backward areas, thereby narrowing the gap between urban and rural areas and regions in basic public health services and accelerating the process of equalization of basic public health services [7].

It is a major test for the national governance system and governance capacity to fight against the COVID-19 epidemic. In view of the shortcomings and deficiencies exposed by the epidemic,we should sum up the experience and learn from the lessons to tighten up the shortcomings, plug the loopholes and strengthen the weaknesses. The purpose of this study is to investigate the difficulties encountered by public health workers in the early and middle stages of the COVID-19 epidemic in China and compare between the primary and non-primary levels. This study will identify shortcomings in epidemic prevention and control, and to provide scientific evidences for improving the national public health emergency management system.

## Materials and methods

### Study design

This study was a cross-sectional study design using a self-administered questionnaire from February 18 to March 1, 2020 in five provinces including Hubei, Guangdong, Sichuan, Jiangsu and Gansu. These five provinces are located in Central China, South China, West China, East China and North China, with a cumulative number of confirmed cases (as of February 25) of 65,187, 1,347, 531, 631 and 91, respectively. The geographic locations of the five provinces were showed in Figure S1. The study was conducted using multi-stage targeted sampling (according to the severity of the epidemic and urban/rural distribution), with 3–6 cities within each province, 3–6 districts and 5–12 streets or communes within each city. In the sampled administrative areas, the staff from the CDCs and HECs at provincial, city and district levels and primary health care institutions (including community health centers/stations, township health centers, and village health clinics) involved in the prevention and control of COVID-19 epidemic were surveyed for this study. The relationship bewteen different levels and types health institutes (e.g., CDC, HEC and CHC) in China were showed in Figure S2. We classified the surveyed institutions into 3 categories: CDCs and HECs (defined as “non-primary”), community health centers/stations in urban (defined as “primary-urban”) and township health centers and village health clinics (defined as “primary-rural”).

### Study subjects

Inclusion criteria for the subjects of this study: (1) age 18 years and above; (2) public health workers involved in work related to COVID-19 outbreak prevention and control. This study distributed a web link containing the questionnaire via WeChat and QQ instant messenger. With the rigorous checking rules of network survey, the questionnaires we received were all valid. However, the response rate could not be computed as the population got the web link were unclear. All respondents were informed prior to the survey of the background, purpose, anonymity, and time required for the survey (approximately 8–12 min). A self-administered questionnaire was administered with the consent of the survey respondents. This study was approved by the Ethics Committee of the School of Public Health, Sun Yat-sen University (No. 2020-012).

### Survey content

This study investigated demographic characteristics, epidemic prevention efforts, health status, encountered difficulties, perceptions of the epidemic, and emotional psychology. The questionnaire was self-designed through literature reviews, in-depth interviews, and focus-group sessions (Table S1). The elements used in this study are demographic characteristics, encountered difficulties and psychological distress. Demographic characteristics include 5 pieces of information: age, gender, child status (presence or absence of children and age of youngest child), job title, and type of institution (primary-rural, primary-urban, and non-primary). The encountered difficulties also included 5 dimensions: resources shortage (5 entries), data processing (5 entries), communication and coordination (4 entries), target audience (3 entries), and psychological distress (4 entries). The encountered difficulties dimensions were multiple-choice except for the psychological distress dimension, which used a 5-point Likert scale (the higher the score, the more severe the situation). We evaluated each dimension comprehensively using bicategorical variables, with the psychological distress dimension set to 1 (more distress) if the mean score was 2.5 (half of the maximum score of 5) or higher, and 0 otherwise, and the other dimension set to 1 (more difficulties) if half or more of the number of items were checked, and 0 otherwise.

### Statistical analysis

The institution type was used as a grouping variable. Continuous variables (i.e., age) were described as mean ± standard deviation (*Mean* ± *SD*), and ANOVA was used for between-group comparisons. Qualitative variables were described as frequencies (proportions or rates) and compared between groups using Fisher’s exact test. In the comprehensive analysis of encountered difficulties, logistic regression was used with bicategorical variables as dependent variable, institution type as independent variable, and the demographic characteristics as covariates. In addtion, we used the generalized linear mixed-effects model including the province as the random effect to exclude the potential variance among the provinces. All the analyses were performed at *P* < 0.05 to indicate statistical significance.

## Results

### Basic characteristics of study subjects

A total of 9475 questionnaires were collected in this study, all of which were valid. Among them, 3786(40.0 %) were from primary-rural, 2561(27.0 %) from primary-urban and 3128(33.0 %) from non-primary. The average age was 38.7 years, with primary-rural being the oldest (39.9 years) and primary-urban being the youngest (36.4 years). The average percentage of females was 64.4 %, with the highest percentage in primary-rural (78.25 %) and the lowest percentage in non-primary (58.12 %). The average percentage of those without children or with children at primary level and below was 63.73 %, with the highest percentage in primary-urban (77.20 %) and the lowest in primary-rural (53.01 %). The average percentage of primary and below titles was 61.45 %, with the highest in primary-rural (77.79 %) and the lowest in non-primary (45.62 %). The differences in the above variables among the three groups were statistically significant (all *P* < 0.001). (Table 1)

**Table 1:** Basic characteristics of the study subjects

	Total	Primary-rural	Primary-urban	Non-primary	*P* value
**Sample size**	9475	3786	2561	3128	
**Age (y)**	38.656 ± 9.704	39.891 ± 10.123	36.381 ± 9.031	39.022 ± 9.395	< 0.001
**Sex**					
Male	3378 (35.65)	1511 (39.91)	557 (21.75)	1310 (41.88)	< 0.001
Female	6097 (64.35)	2275 (60.09)	2004 (78.25)	1818 (58.12)	
**Child status**					
Absence	2161 (22.81)	621 (16.40)	776 (30.30)	764 (24.42)	< 0.001
Primary school and below	3877 (40.92)	1386 (36.61)	1201 (46.90)	1290 (41.24)	
Junior high school and above	3437 (36.27)	1779 (46.99)	584 (22.80)	1074 (34.34)	
**Job title**					
Primary and below	5822 (61.45)	2945 (77.79)	1450 (56.62)	1427 (45.62)	< 0.001
Intermediate	2682 (28.31)	718 (18.96)	930 (36.31)	1034 (33.06)	
Advanced	971 (10.25)	123 (3.25)	181 (7.07)	667 (21.32)	
**Province**					< 0.001
Sichuan	3360 (35.46)	1812 (47.86)	610 (23.82)	938 (29.99)	
Guangdong	2812 (29.68)	455 (12.02)	1559 (60.87)	798 (25.51)	
Jiangsu	1227 (12.95)	564 (14.90)	193 (7.54)	470 (15.03)	
Hubei	1401 (14.79)	893 (23.59)	135 (5.27)	373 (11.92)	
Gansu	675 (7.12)	62 (1.64)	64 (2.50)	549 (17.55)	

Note: Continuous variables are expressed as mean ± standard deviation and categorical variables are expressed as frequencies (proportions)

### Resources shortage

We found that public health workers encounter multiple resource deficiencies in their work. Resources shortage were reported for protective gear (87.4 %), own skills (38.5 %), manpower (47.1 %), funding (20.3 %) and reagents (8.0 %). In particular, non-primary had a higher rate of reporting inadequate manpower and reagents than the primary (all *P* < 0.01), and conversely, the primary had a higher rate of reporting inadequate protective equipment (*P* < 0.001). Comparing the two types of the primary institutions found that primary-urban had a higher rate of reporting inadequate protective equipment (*P* = 0.009), while primary-rural had a higher rate of reporting inadequate own skills, manpower, funding and reagents (all *P* < 0.01). Among the protective equipment, deficiencies were reported, in descending order, for N95 masks (80.5 %), medical surgical masks (80.4 %), protective clothing (77.5 %), medical goggles (57.0 %), medical alcohol (47.9 %), forehead thermometers (43.6 %) and gloves (37.3 %). (Table 2)

**Table 2:** Resources shortage between primary and non-primary institutions

	Total *n*(%)	Primary- rural (%)	Primary- urban (%)	Non- primary (%)	*P* value	Primary vs. Non-primary *P* value*	Primary-rural vs. Primary-urban *P* value*
**Protective equipment**	8280 (87.39)	90.62	92.50	79.28	< 0.001	< 0.001	0.009
**Self-skill**	3645 (38.47)	43.16	28.47	40.98	< 0.001	< 0.001	< 0.001
**Manpower**	4459 (47.06)	45.69	41.66	53.13	< 0.001	< 0.001	0.002
**Funding**	1927 (20.34)	24.64	14.76	19.69	< 0.001	0.278	< 0.001
**Reagents**	761 (8.03)	8.80	5.23	9.40	< 0.001	0.001	< 0.001
**Protective equipment**							
N95 mask	6663 (80.49)	76.61	80.71	85.65	< 0.001	< 0.001	< 0.001
Surgical masks for medical use	6654 (80.38)	81.54	81.43	77.78	0.001	< 0.001	0.918
Protective clothing	6419 (77.54)	76.03	82.57	74.84	< 0.001	< 0.001	< 0.001
Medical goggles	4714 (56.95)	63.43	58.08	46.90	< 0.001	< 0.001	< 0.001
Medical alcohol	3964 (47.89)	53.40	46.01	42.06	< 0.001	< 0.001	< 0.001
Epidural gun	3611 (43.62)	56.87	38.88	29.84	< 0.001	< 0.001	< 0.001
Medical gloves	3088 (37.30)	44.94	35.46	28.51	< 0.001	< 0.001	< 0.001

*The *P* values were corrected for multiple comparisons. “Total” column was presented as frequency (reporting rate). “Primary-rural”, “Primary-urban”, and “Non-primary” columns were presented as reporting rates

### Data processing

We found that public health workers encountered difficulties in data processing in their work. The reported encountered difficulties in data processing were: excessive documentation (63.8 %), cumbersome and time-consuming data filling (49.8 %), cumbersome and time-consuming work accounts (36.9 %), time-consuming transmission of information (25.7 %) and inconvenient transmission of documents (14.4 %). Among them, non-primary had a higher rate of reporting encountered difficulties such as cumbersome and time-consuming data filling, time-consuming information reporting and inconvenient document information transmission than the primary (all *P* < 0.01). Comparing the two types of primary institutions found no statistical difference in the reported rate of encountered difficulties in data processing. (Table 3)

**Table 3:** Difficulties in data processing, communication and coordination, and target audiences between primary and non-primary institutions

	Total *n*(%)	Primary- rural (%)	Primary- urban (%)	Non- primary (%)	*P* value	Primary vs. Non-primary *P* value*	Primary-rural vs. Primary-urban *P* value*
**Data processing**							
Excessive documentation	6040 (63.75)	63.63	63.84	63.81	0.982	0.946	0.946
Cumbersome and time-consuming data filling	4720 (49.82)	47.99	49.39	52.37	0.002	0.001	0.282
Cumbersome and time-consuming work accounts	3500 (36.94)	37.64	35.85	36.99	0.362	0.946	0.305
Time-consuming transmission of information	2433 (25.68)	23.77	22.10	30.91	< 0.001	< 0.001	0.122
Inconvenient transmission of documents	1367 (14.43)	12.86	12.26	18.09	< 0.001	< 0.001	0.488
**Communication and coordination**							
Poor inter-agency coordination	3346 (35.31)	26.70	36.70	44.60	< 0.001	< 0.001	< 0.001
Poor intra-departmental coordination	2091 (22.07)	19.31	18.74	28.13	< 0.001	< 0.001	0.580
Unclear assignments from superiors	1740 (18.36)	14.53	15.97	24.97	< 0.001	< 0.001	0.116
Unclear overtime incentive system	3851 (40.64)	36.90	36.78	48.34	< 0.001	< 0.001	0.937
**Target audiences**							
Uncooperative	3800 (40.11)	39.28	46.47	35.90	< 0.001	< 0.001	< 0.001
Verbal abuse/intimidation by work targets	1297 (13.69)	12.57	18.00	11.51	< 0.001	< 0.001	< 0.001
Concerns about survey reliability	3402 (35.91)	34.57	35.38	37.95	0.019	0.008	0.520

*The *P* values were corrected for multiple comparisons. “Total” column was presented as frequency (reporting rate). “Primary-rural”, “Primary-urban”, and “Non-primary” columns were presented as reporting rates

### Communication and coordination

We found that public health workers encounter a variety of hard situations in their communication and coordination work. Difficulties in communication and coordination were reported as poor inter-agency coordination (35.3 %), poor intra-departmental coordination (28.1 %), unclear assignments from superiors (18.4 %), and unclear overtime incentive system (40.6 %). Of these, all of the above encountered difficulties were higher reported by non-primary compared with the primary-rural and primary-urban (all *P* < 0.001). Comparing the two types of primary institutions found that primary-urban had a higher rate of reporting encountered difficulties with poor intra-departmental coordination (*P* < 0.001). (Table 3)

### Target audience

We found that outbreak prevention and control workers encounter a variety of hard situations with the target audience they work with. The rates of reported difficulties with target audience were: uncooperative (40.1 %), verbal abuse/intimidation by work targets (13.7 %), and concerns about survey reliability (36.0 %). Of these, non-primary had a higher rate of reporting concerns about survey reliability than the primary (*P* < 0.001). Comparing the two types of primary institutions found that primary-urban had a higher rate of reporting difficulties with uncooperative and verbal abuse/intimidation by work targets (*P* < 0.001). (Table 3)

### Psychological distress

We found that the public health workers encountered multiple situations of psychological distress in their work. The levels of each type of psychological distress were: being treated differently at work (2.4 points), feeling aggrieved at work (2.6 points), family members not understanding (1.9 points) and worrying about routine work outside of the epidemic (2.6 points). Among them, non-primary had higher levles of being treated differently at work and feeling aggrieved at work than the primary (both *P* < 0.001). While the primary had higher levels of worrying about routine work outside of the epidemic (*P* = 0.003). Comparing the two types of primary institutions found that primary-urban had a higher rate of reporting distress with family members not understanding (*P* = 0.002), and primary-rural had a higher rate of reporting distress with worrying about routine work outside of the epidemic (*P* = 0.003). (Table 4)

**Table 4:** Psychological distress between primary and non-primary institutions

	Total *M* ± *SD*	Primary- rural *M* ± *SD*	Primary- urban *M* ± *SD*	Non- primary *M* ± *SD*	*P* value	Primary vs. Non-primary *P* value*	Primary-rural vs. Primary-urban *P* value*
Being treated differently at work	2.437 ± 0.984	2.395 ± 1.002	2.383 ± 0.909	2.527 ± 1.013	< 0.001	< 0.001	0.741
Feeling aggrieved at work	2.572 ± 1.003	2.539 ± 1.013	2.506 ± 0.945	2.665 ± 1.029	< 0.001	< 0.001	0.405
Family members not understanding	1.867 ± 0.883	1.849 ± 0.904	1.903 ± 0.867	1.857 ± 0.870	0.046	0.654	0.002
Worrying about routine work outside of the epidemic	2.640 ± 0.990	2.692 ± 0.999	2.608 ± 0.963	2.604 ± 0.999	< 0.001	0.003	0.003

* The *P* values were corrected for multiple comparisons. M ± SD indicates mean ± standard deviation

### Comprehensive analysis

All five dimensions of encountered difficulties were reported at a relative high rate (20.2-48.8 %). The difference in reporting rates were statistically significant among institutions for resources shortage, coordination and communication, and target audience. Of these, resources shortage was more frequently reported in primary-rural (*OR* = 1.201, 95 %*CI*: 1.073–1.345); Difficulties in communication and coordination in non-primary were reported at higher rates (primary-rural: *OR* = 0.520, 95 %*CI*: 0.446–0.606, primary-urban: *OR* = 0.533, 95 %*CI*: 0.454–0.625); Primary-rural reported higher rates in difficulties with target audience (*OR* = 1.368, 95 %*CI*: 1.199–1.560). Consistent results were obtianed from the generalized linear mixed-effects models. (Table 5)

**Table 5:** Comparative analysis of the five dimensions of encountered difficulties between primary and non-primary institutions

	Resources shortage *OR*(95 %CI)	Data processing *OR*(95 %CI)	Communication and coordination *OR*(95 %CI)	Target audiences *OR*(95 %CI)	Psychological distress *OR*(95 %CI)
**Institution, reporting rate (%)**					
Total	27.9	31.5	29.8	20.2	48.8
Non-primary	28.8	34.1	40.9	19.6	50.7
Primary-rural	32.1	30.6	22.8	18.4	48.0
Primary-urban	20.7	29.6	26.8	23.4	47.5
**Unadjusted**					
Institution (ref.= Non-primary)					
Primary-rural	1.171(1.056,1.298)**	0.850(0.768,0.940)**	0.389(0.338,0.448)***	0.927(0.822,1.046)	0.899(0.818,0.988)*
Primary-urban	0.643(0.569,0.728)***	0.812(0.726,0.909)***	0.458(0.393,0.534)***	1.253(1.103,1.422)**	0.880(0.793,0.977)*
**Adjusted 1**^a^					
Institution (ref.= Non-primary)					
Primary-rural	1.201(1.073,1.345)**	1.010(0.904,1.129)	0.520(0.446,0.606)***	1.085(0.952,1.237)	0.996(0.898,1.104)
Primary-urban	0.713(0.629,0.810)***	0.916(0.815,1.029)	0.533(0.454,0.625)***	1.368(1.199,1.560)**	0.901(0.809,1.003)
**Adjusted 2**^b^					
Institution (ref.= Non-primary)					
Primary-rural	1.311(1.163,1.478)***	0.990(0.882,1.111)	0.596(0.529,0.671)***	1.146(0.999,1.315)	1.068(0.958,1.190)
Primary-urban	0.728(0.636,0.833)***	0.871(0.769,0.987)*	0.560(0.494,0.635)***	1.282(1.114,1.475)**	0.924(0.824,1.037)

^a^The variables including age, gender, child status and job title were adjusted

^b^The variables including age, gender, child status and job title were adjusted, as well as the province was included as a random effect

*P < 0.05, **P < 0.01, ***P < 0.001

## Discussions

This study, based on a survey of 9,475 public health workers, explored possible shortcomings in the early and middle stages of major outbreak prevention and control work, and found that the highest reporting rate was psychological distress (48.8 %), the middle reporting rate was data processing (31.25 %), communication and coordination (29.8 %) and resource shortage (27.9 %), and the lowest reporting rate was target audience (20.2 %). A comparative analysis among different institutions found higher rates of resources shortage in primary-rural, higher rates of communication and coordination in non-primary, and higher rates of target audience in primary-urban.

The mental health problems of public health workers cannot be ignored. The psychological distress was found as the most serious in this study, with a reporting rate of nearly half (48.8 %). And there was no variation among institutions, suggesting that despite the differences in the content of outbreak prevention and control work in different institutions, all faced high levels of psychological distress. Mental health problems of clinical staff in outbreak prevention and control have raised concern [8]. This study found that the mental health problems of public health workers are also of concern, and further explored their specific sources of distress, such as: being treated differently at work, feeling aggrieved at work, family members not understanding and worrying about routine work outside the epidemic. Li et al. found a higher reported rate of depression (21.3 %) and anxiety (19.0 %) among the public health workers in COVID-19 epidemic [9]. The mental health impact of the first wave of COVID-19 pandemic on Spanish healthcare workers were also reported with a prevalence of 28.1 % in major depressive Dissorder [10]. Similarly, 28.4 % healthcare workers in Spain were found with a medium–high emotional load or extreme acute stress [11]. These psychological distresses directly affect epidemic prevention on the one hand, and pose mental health hazards to staff on the other. In the future, the comprehensive protection of public health workers should be improved [12], and humanistic care should be strengthened so that they can work with peace of mind and efficiency [13]. In addition, the current psychological intervention for epidemic workers focuses on clinical health care staff, and the psychological relief and intervention for public health workers cannot be ignored.

The problem of resources shortage in primary-rural is of concern. The resources shortage problem found in this study is serious (27.9 % reporting rate) and the highest reporting rate in primary-rural, suggesting that resources allocation in primary-rural needs to be optimized in the early and middle stages of epidemic. Moreover, a previous study showed that at least 70 % healthcare workers reported a lack of personal protective equipment including gown coverall suits, N95 masks, and face shields [14]. Admittedly, the resources shortage in China occurred mainly in the early stage of epidemic. Because with the development of the epidemic, the capacity and transfer of epidemic prevention materials work in an orderly manner, which gradually ensure that China’s reserve materials are sufficient. Resources shortage was mainly manifested in insufficient emergency material reserves and the capacity of health emergency response teams needs to be improved. Government financial support for health emergencies should be increased and the allocation of resources for health emergencies optimized. Vulnerable areas with relatively insufficient financial support (e.g., the primary-rural identified in this study) are often the focus of health emergency work. The introduction, training and training of health emergency staff should be strengthened, and drills and training in on-site epidemiological investigation are important ways to improve health emergency response capacity [15].

Difficulties in communication and coordination in non-primary institutions need attention. The difficulties in communication and coordination found in this study are serious (the reporting rate is 29.8 %), and the highest reporting rate is found in non-primary, suggesting that the reform of China’s CDC institutions needs to pay attention to information communication and transportation coordination. As the core backbone of the public health network, the work of CDC institutions involves more communication and coordination. Difficulties in communication and coordination are mainly manifested in unclear overtime incentive system and poor communication between and within departments, which will directly affect the implementation of epidemic prevention and control and its effectiveness. To address difficulties in communication and coordination, we should put the staff overtime incentive performance programs [16] and the multi-departmental joint prevention and control mechanisms at different levels [17] into the construction of the emergency system for public health emergencies.

Difficulties with target audiences in primary-urban were relatively prominent. The difficulties with target audiences found in this study were relatively serious (the reporting rate is 20.2 %), and the reporting rate is highest in primary-urban, suggesting that the focus of public education on public health in China should be on primary-urban. Difficulties with target audiences are mainly manifested in concerns about the reliability of the survey and the uncooperative, which will directly affect the effect of blocking the transmission route and protecting vulnerable groups in epidemic prevention and control. In order to address the difficulties with target audiences, vigorous efforts should be made to promote community awareness of public health and emergency work, so that the community can understand the relevant work, reduce misunderstandings and promote prevention and control work. For key populations with a lower level of education, we should use appropriate methods to publicize and popularize core information on the prevention and control of infectious diseases, cultivate their good hygiene habits and healthy lifestyles, and raise the overall population’s awareness of the prevention and control of infectious diseases [18].

There are limitations to this study. First, the multi-stage sampling according to geographic distribution and the severity of the epidemic may be subject to selection bias, leading to an increased risk of extrapolating the findings to other parts of the country. Second, this study used a self-administered questionnaire, which may be subject to reporting bias. Third, this study was a cross-sectional survey and it cannot yet be assumed that the shortcomings identified were only in the context of preventing and controlling the COVID-19 epidemic.

## Conclusions

In summary, China’s epidemic prevention and control personnel have played an important role in the fight against the COVID-19 epidemic. We found that the most serious in the prevention and control work was psychological distress. The resources shortage in primary-rural, the difficulties in communication and coordination in non-primary, and the difficulties with target audiences in primary-urban were worthy of attention. This study will provide a scientific basis for improving the national public health emergency management system, especially for reducing the urban-rural disparity in emergency response capacity.

## Supplementary Information

**Additional file 1.**

## Data Availability

The datasets during and/or analysed during the current study available from the corresponding author on reasonable request.
