# Supplementary material for: Difficulties encountered by public health workers in COVID-19 outbreak: a cross-sectional study based on five provinces
Source: BMC Health Serv Res. 2021 Jul 5;21:656. doi: 10.1186/s12913-021-06699-4 (PMC8256394; doi:10.1186/s12913-021-06699-4)
Supplement: Supplementary file 1 — Additional file 1. [file 12913_2021_6699_MOESM1_ESM.docx]

**Supplementary materials**

**Difficulties encountered by public health workers in COVID-19 outbreak: a cross-sectional study based on five provinces**

Zhicheng Du ^1#^, Hua You ^2#^, Huan Zhou ^3^, Xiaohui Wang ^4^, Jingdong Xu ^5^, Yan Li ^6^, Shan Li ^7^, Lina Ma ^5^, Jing Gu ^1*^, Yuantao Hao ^1^

^1^School of Public Health, Global Health Institute, Sun Yat-sen University; Key Laboratory of Tropical Disease Control (Sun Yat-sen University), Ministry of Education, Guangzhou 510080

^2^School of Public Health, Nanjing Medical University, Nanjing 210000

^3^West China School of Public Health, No. 4 West China Teaching Hospital, Sichuan University, Chengdu 610000

^4^School of Public Health, Lanzhou University, Lanzhou 730000

^5^Hubei Center for Disease Control and Prevention, Wuhan 430097

^6^Guangzhou Center for Disease Control and Prevention, Guangzhou 510440

^7^Zigong Center for Disease Control and Prevention, Zigong 643000

^#^Zhicheng Du and Hua You have equal contributions

^*^Corresponding author: Jing Gu, Email: gujing5@mail.sysu.edu.cn

**Table S1. Questionnaire on difficulties encountered by public health workers in COVID-19 outbreak.**

| **1. Basic Information** |
| --- |
| 1.1 Sex: □Male □Female |
| 1.2 Age: ____ years |
| 1.3 Do you have children? □No □Yes  if yes: How old is your youngest child? □Primary school and below □Junior high school and above |
| 1.4 What’s your job title? □Primary and below □Intermediate □Advanced |
|  |
| **2. What difficulties have you encountered in your work on the epidemic?** |
| 2.1 Resources shortage (multiple choice): □Protective equipment □Self-skill □Manpower □Funding □Reagents  if “Protective equipment” (multiple choice): □N95 mask □Surgical masks for medical use □Protective clothing □Medical goggles □Medical alcohol □Epidural gun □Medical gloves |
| 2.2 Data processing (multiple choice): □Excessive documentation □Cumbersome and time-consuming data filling □Cumbersome and time-consuming work accounts □Time-consuming transmission of information □Inconvenient transmission of documents |
| 2.3 Communication and coordination (multiple choice): □Poor inter-agency coordination □Poor intra-departmental coordination □Unclear assignments from superiors □Unclear overtime incentive system |
| 2.4 Target audiences (multiple choice): □Uncooperative □Verbal abuse/intimidation by work targets □Concerns about survey reliability |
|  |
| **3. Do you have any trouble with the following aspects of your work on the epidemic? To what extent?** |
| 3.1 Being treated differently at work: □None □Few □Medium □Often □Very often |
| 3.2 Feeling aggrieved at work: □None □Few □Medium □Often □Very often |
| 3.3 Family members not understanding: □None □Few □Medium □Often □Very often |
| 3.4 Worrying about routine work outside of the epidemic: □None □Few □Medium □Often □Very often |


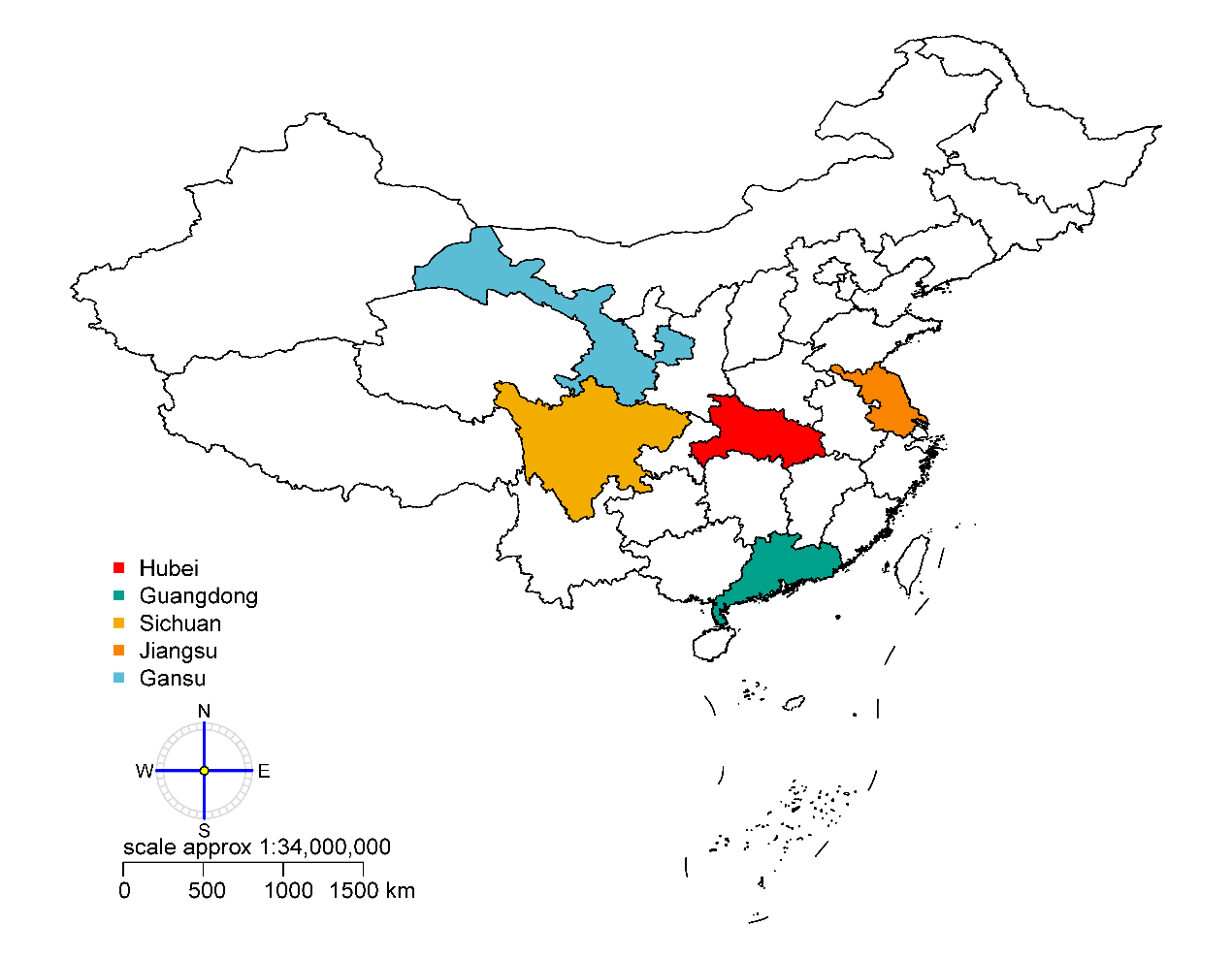


**Figure S1. The geographic locations of the five provinces.**


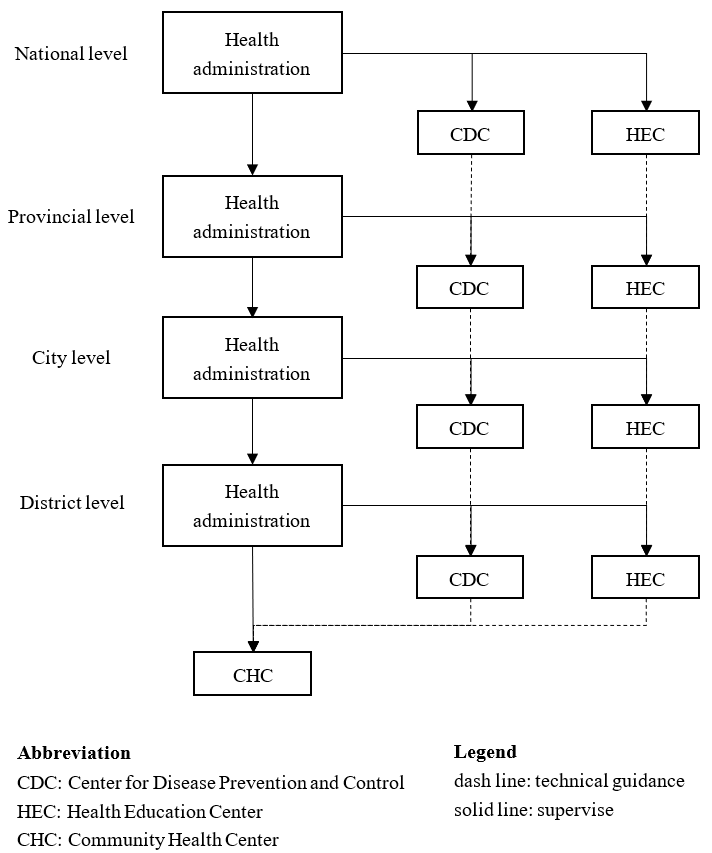


**Figure S2. The relationship bewteen different levels and types health institutes in China.**
